# Approach to Low Contact Resistance Formation on Buried Interface in Oxide Thin-Film Transistors: Utilization of Palladium-Mediated Hydrogen Pathway

**DOI:** 10.1021/acsnano.4c02101

**Published:** 2024-03-22

**Authors:** Yuhao Shi, Masatake Tsuji, Hanjun Cho, Shigenori Ueda, Junghwan Kim, Hideo Hosono

**Affiliations:** 1 MDX Research Center for Element Strategy, International Research Frontiers Initiative, Tokyo Institute of Technology, Yokohama 226-8503, Japan; 2 Research Center for Electronic and Optical Materials, National Institute for Materials Science (NIMS), Tsukuba, Ibaraki 305-0044, Japan; 3 Graduate School of Semiconductor Materials and Devices Engineering, Ulsan National Institute of Science and Technology, Ulsan 44919, Republic of Korea; 4 Research Center for Materials Nanoarchitectonics, NIMS, Tsukuba, Ibaraki 305-0044, Japan

**Keywords:** oxide semiconductors, IGZO, thin-film transistors, contact resistance, palladium, hydrogen, bottom contact

## Abstract

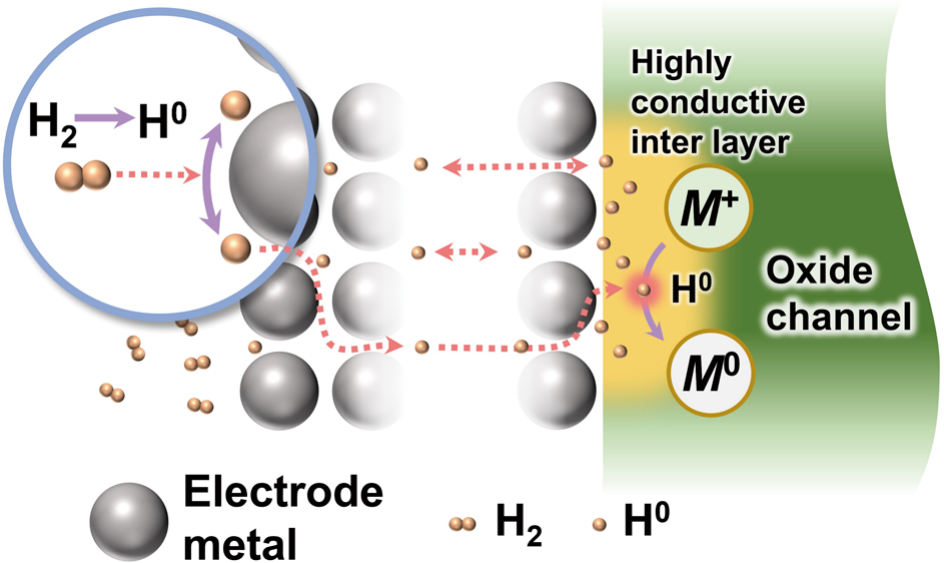

Amorphous oxide semiconductors (AOSs) with low off-currents and processing temperatures offer promising alternative materials for next-generation high-density memory devices. The complex vertical stacking process of memory devices significantly increases the probability of encountering internal contact issues. Conventional surface treatment methods developed for planar devices necessitate efficient approaches to eliminate contact issues at deep internal interfaces in the nanoscale complex structures of AOS devices. In this work, we report the pioneering use of palladium thin film as a high-efficiency active hydrogen transfer pathway from the outside to the internal contact interface via low-temperature postannealing in the H_2_ atmosphere, and the formation of highly conductive metallic interlayer effectively solves the contact issues at the deeply buried interfaces in devices. The application of this method reduced the contact resistance of Pd electrodes/amorphous indium–gallium–zinc oxide (a-IGZO) thin-film by 2 orders of magnitude, and thereby the mobility of thin-film transistor was increased from 3.2 cm^2^ V^–1^ s^–1^ to nearly 20 cm^2^ V^–1^ s^–1^, preserving an excellent bias stress stability. This technology has wide applicability for the solution of contact resistance issues in oxide semiconductor devices with complex architectures.

## Introduction

**Figure 1:**
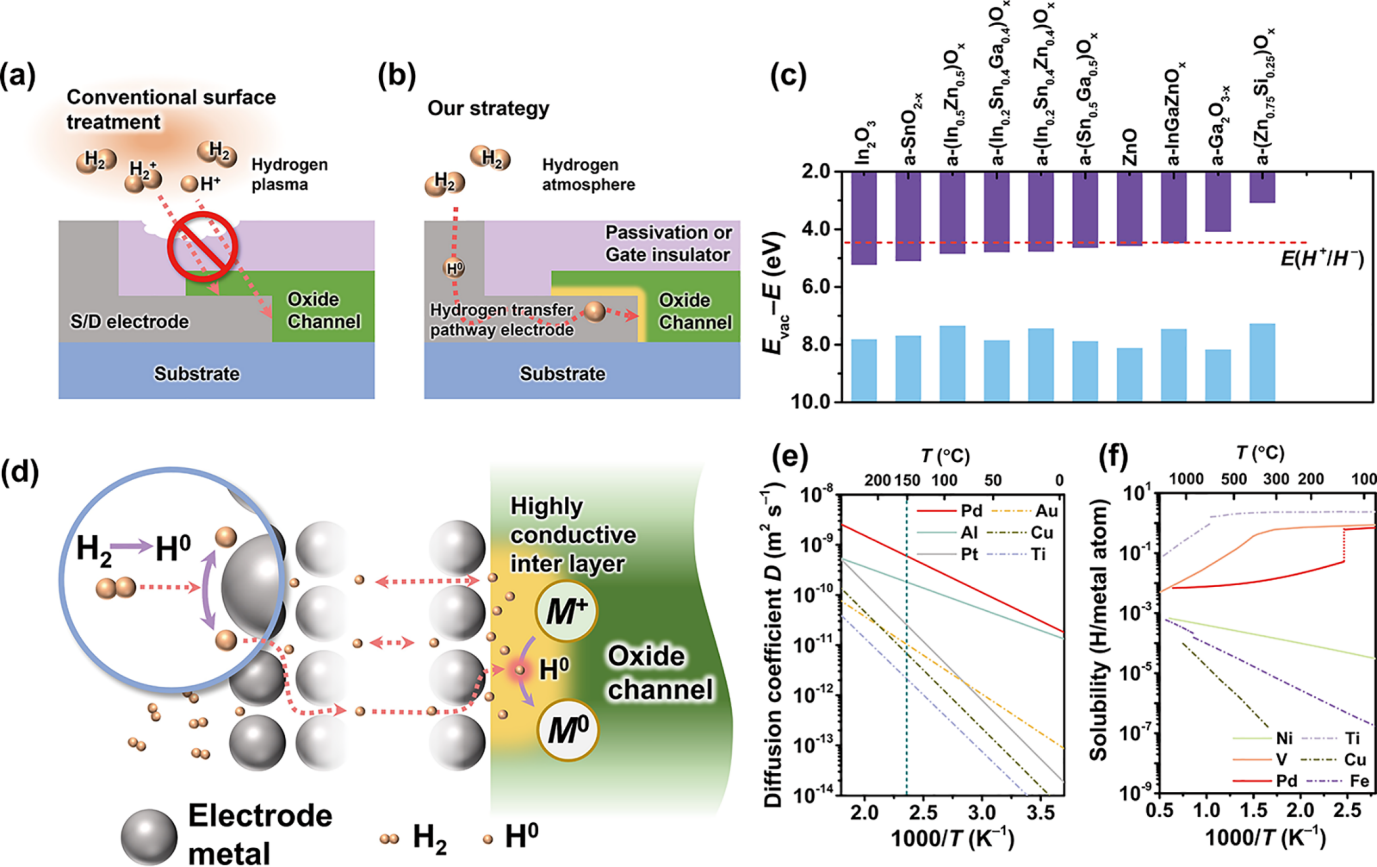
Strategy to solve the contact issue of the internal contact interface. Schematic diagram of (a) hydrogen plasma surface treatment and (b) hydrogen transfer pathway method for bottom-contact oxide TFTs. (c) Energy levels of valence band maximum (*E*_VBM_) and *E*_CBM_ in oxide semiconductors with a hydrogen charge transmission level (4.5 eV).^4,27,28^ (d) Schematic diagram of catalytic hydrogen dissociation at the electrode metal surface and reaction between highly active atomic hydrogens and oxide. (e) Experimental values for hydrogen diffusion in Pd,^32^ Al,^33^ Pt,^34^ Au,^34^ Cu,^35^ and Ti.^36^ (f) Hydrogen solubility based on hydrogen-to-metal atom ratio in Ti, V, Pd, Ni, Fe, and Cu.^37^

Amorphous oxide semiconductors (AOSs) with extremely low off-currents originating from their electronic structure^1−4^ have attracted considerable interest for applications in the storage chip industry, enabling the development of capacitorless dynamic random-access memory (DRAM) architecture and high-density DRAM technologies.^5−8^ In contrast to thin-film transistors (TFTs) for flat panel displays, storage chips employ vertically stacked complex device architectures to achieve higher device density,^9−11^ posing challenges in electrode processing, and increasing the importance of contact issues between AOSs and electrodes.^12−14^ Process-derived poor contacts and a substantial Schottky barrier resulting from the intrinsic energy level mismatch between the work function of the electrode metal and the electron affinity of AOSs eventually lead to excessively high contact resistance (*R*_C_), thereby degrading field-effect mobility and power consumption. Recently, many works have proposed methods to solve high contact resistance between AOSs and metal electrodes, which may be categorized into several main strategies: additional deposition of a highly conductive oxide interlayer,^15−17^ oxidation of the metal contact surface resulting in the formation of high concentration oxygen vacancies on the AOS contact surface via high-temperature annealing,^18−21^ penetration of metal ions into the AOS layer,^22,23^ and surface treatment with plasma.^24−26^ These methods, which involve high-energy or multistep processes, offer effective solutions for the high contact resistance of the exposed upper surface of oxide semiconductors, as shown in Figure 1a, but are almost impossible to apply to buried contact or deep vertical interfaces within nanoscale complex structures.

Hydrogen is a crucial and influential element in oxide semiconductors during thin-film deposition and post-treatment.^4^Figure 1c provides a summary of the band-edge energy of various oxide semiconductor materials compared to the universal charge transition level of *E*(H^+^/H^–^).^4,27,28^ When the conduction band minimum (*E*_CBM_) of an oxide semiconductor is lower than *E*(H^+^/H^–^), such as amorphous indium–gallium–zinc oxide (a-IGZO), the hydrogen injected into the oxide ionizes to generate carrier electrons (e^–^), and the resulting H^+^ is stabilized in the form of OH, increasing the carrier concentration.^4,29,30^ Consequently, the injection/implantation of hydrogen into AOS thin films contacting the electrode area has emerged as an effective method for the formation of a highly conductive oxide interlayer, thereby mitigating the contact resistance between AOSs and metal electrodes. Hydrogen plasma is a conventional approach to address contact issues, where H_2_ is dissociated into H^+^ and an electron, followed by the treatment of the exposed oxide surface, which forms a highly conductive contact interface. However, hydrogen plasma is not a suitable solution for internal contact interfaces covered by a passivation layer or a gate insulator (Figure 1a). To ensure effective and precise action of hydrogen at an internal interface such as passivated bottom-contact TFTs (Figure 1b), we think employing a metal material capable of transporting hydrogen as the electrode and serving as a hydrogen transfer pathway is an effective strategy. Furthermore, single atomic H^0^, compared to H_2_, exhibits a much stronger reduction power to metal oxides at low temperatures,^31^ facilitating more efficient interface metallization. Figure 1d shows a strategy based on the quest for a metal electrode material exhibiting the capacity for catalytically dissociating hydrogen molecules into highly reactive atomic H^0^ at low temperatures to adhere to the temperature limitations of the semiconductor fabrication process. The hydrogen diffusion rate and hydrogen solubility of the chosen metal are crucial for hydrogen to rapidly transfer to and efficiently undergo a reduction reaction with the interface oxide at relatively low temperatures. Therefore, choosing electrode materials with catalytic capabilities for dissociating H_2_ into atomic H^0^ and superior hydrogen transport properties is key to achieving this strategy.

Here we focus on the properties of the metal palladium on hydrogen. Pd exhibits a high hydrogen diffusion rate at low temperatures, as shown in Figure 1e, offering an efficient means to shorten post-hydrogen treatment times and reduce processing temperatures. In addition, the moderate hydrogen solubility of Pd (Figure 1f, 0.01–0.6 H/metal atom) is favorable for facile adsorption and desorption of hydrogen.^37,38^ This feature makes it possible for reversible in–out hydrogen species between contact materials (outer environment) and Pd, which is akin to a catalyst and improves the contact efficiency between hydrogen and AOS. Based on its very high hydrogen diffusion rate and smooth in/out feature, one may expect that Pd ensures swift hydrogen transport and the formation of a conductive layer at low temperatures, even at deep internal contact surfaces of the device structure. The former feature originates from the neutral state of hydrogen in Pd because H_2_ is catalytically dissociated into atomic H^0^ on the Pd surface,^39,40^ which is expected to effectively enhance metallization reactions at the interface under low-temperature conditions. The characteristics of Pd align well with the dual requirements of catalyzing the dissociation of H_2_ and efficiently transporting hydrogen, making it the most suitable material for addressing the contact resistance between AOSs and metal electrodes at low temperatures with the H_2_ annealing treatment.

In this work, a technique based on Pd electrodes for hydrogen transfer pathways was developed, effectively addressing the contact issue between a-IGZO and electrodes, particularly at internal contact surfaces within nanoscale device architectures. Because of the catalyst-like hydrogen absorption and release of Pd, along with its high hydrogen diffusion rate at low temperatures, a substantial amount of hydrogen can be transferred via Pd transfer pathways from the 5% hydrogen atmosphere to the internal contact surface with a-IGZO during low-temperature postannealing. At the interface between a-IGZO and Pd, numerous oxygen atoms interact with diffused hydrogen, leading to the generation of subsequent metallization of a-IGZO thin films, as confirmed by secondary ion mass spectrometry (SIMS) and hard X-ray photoelectron spectroscopy (HAXPES), which can probe several nanometers in depth from the top surface. The highly metalized interface reduced the contact resistance between a-IGZO and the metal electrodes from 3 kΩ cm to 6.1 Ω cm and enhanced the field-effect mobility (μ_FE_) of the bottom contact a-IGZO TFT from 3.2 to 18.1 cm^2^ V^–1^ s^–1^. No serious instability for negative bias stress (NBS) and positive bias stress (PBS) at room temperature and high temperature was observed, indicating that there was almost no influence of H diffusion on the channel.

## Results and Discussion

### Contact Resistance

**Figure 2:**
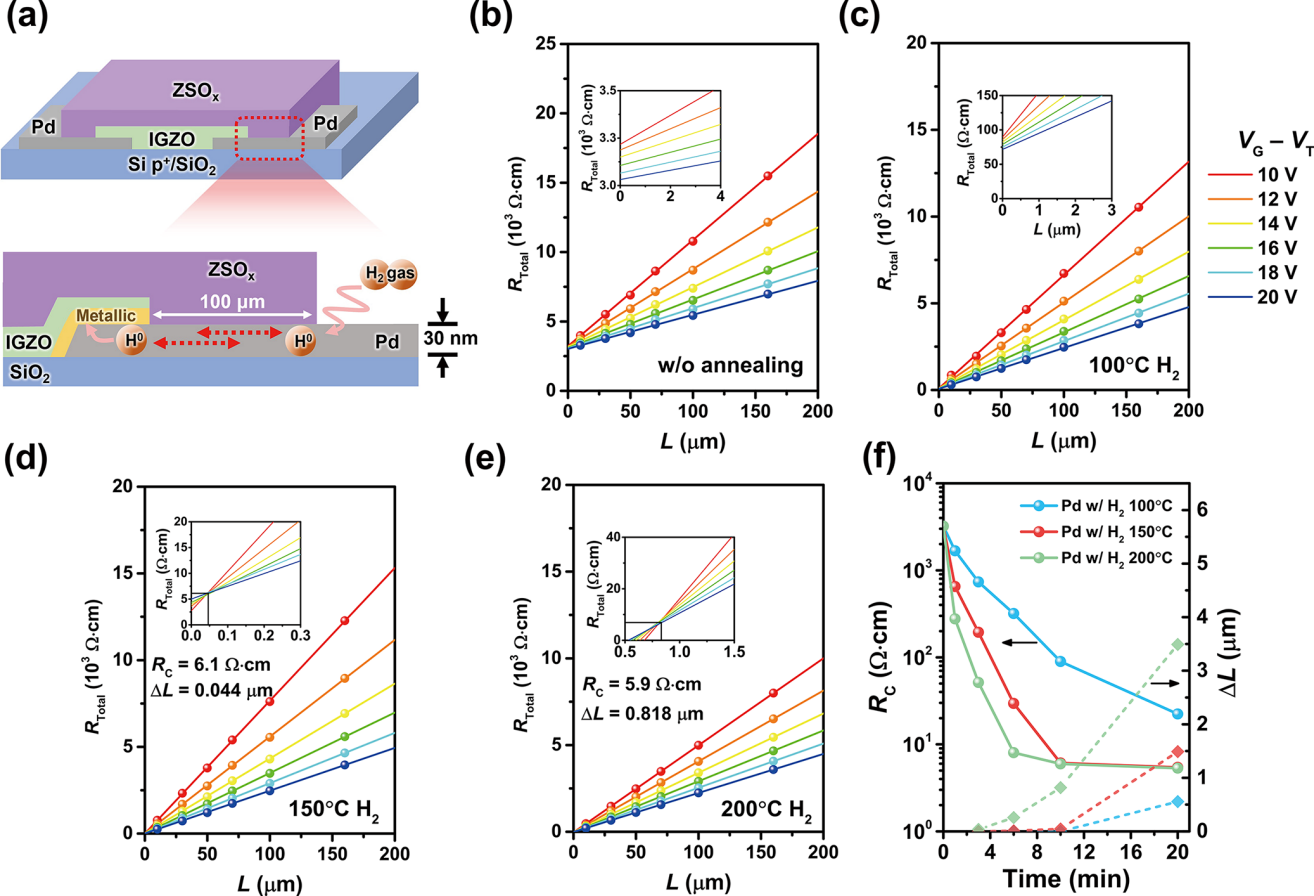
a-IGZO TFT structure and contact resistance. (a) Schematic cross-section of the BGBC a-IGZO TFT passivated by amorphous ZSO_*x*_, and schematic diagram of the Pd-based hydrogen transfer pathway. TLM extractions for contact resistance with Pd electrodes before (b) and after H_2_ annealing treatment at (c) 100 °C, (d) 150 °C, and (e) 200 °C in 10 min (*V*_G_ – *V*_T_ = 10 to 20 V). (f) Contact resistance and channel length deviation (Δ*L*) extracted by the TLM method as a function of annealing time.

Bottom-gate bottom-contact (BGBC) TFT devices were fabricated to demonstrate the feasibility of using Pd thin films as hydrogen transfer pathway electrodes for low-contact resistance (Figure 2a). The 20-nm-thick a-IGZO was utilized as the channel, and the entire device was covered with a 50-nm-thick amorphous ZnO–SiO_2_ (ZSO_*x*_) passivation layer, with only a portion of Pd exposed at both ends of the source and drain electrodes. As a passivation layer, ZSO_*x*_ exhibits a high *E*_CBM_, a higher density, and a flatter surface compared with conventional insulator materials, effectively isolating the impact of impurities and water from the AOS channel.^41^ As shown in Figure 1c, the charge transition level of *E*(H^+^/H^–^) being much lower than the ZSO_*x*_’s *E*_CBM_ ensures that the ZSO_*x*_ passivation layer remains unaffected by H_2_ annealing treatment, thereby modulating carrier concentration in a-IGZO channel by the H_2_-annealing. This design ensures that hydrogen permeates into the channel solely through Pd, preventing diffusion of H to the channel layer from the upper layer. As illustrated in the magnified cross-section in Figure 2a, the constructed electrodes, measuring 100 μm in length and 30 nm in thickness, effectively validate the exceptionally high hydrogen transmission efficiency of Pd at low temperatures. The transmission line measurement (TLM) was employed to extract *R*_C_, using different effective gate biases (*V*_G_ – *V*_T_, where *V*_G_ is the gate voltage, and *V*_T_ is the threshold voltage) ranging from 10 to 20 V. The contact resistance of the as-fabricated devices exceeded 3 × 10^3^ Ω cm as shown in Figure 2b, and the variation of *R*_C_ with *V*_G_ – *V*_T_ indicated the presence of Schottky charge injection between a-IGZO and Pd.^42^ After postannealing was made in a 5% H_2_ atmosphere at 100 °C for 10 min, the contact resistances of the a-IGZO TFTs decreased to below 10^2^ Ω cm (Figure 2c), indicating that the Pd-based electrodes effectively transferred hydrogen from the annealing atmosphere to the internal contact surface at 100 °C. Specifically, the diffused hydrogen during the H_2_ annealing treatment resulted in the reaction with oxygen in the a-IGZO channel, generating electron carriers via oxygen vacancy formation at the interface side and, in turn, weakening the interface Schottky barrier contact via the enhancement of the tunneling contribution. As the H_2_ annealing temperature increased to 150 °C, the *R*_C_ of the a-IGZO TFT decreased by 1 order of magnitude to 6.1 Ω cm (Figure 2d), accompanied by a small reduction in the effective channel length (Δ*L* = 0.044 μm). The decrease in *R*_C_ and the decrease in the effective channel length indicate ohmic contact and the formation of a high-electron-density interfacial layer at the contact surface. The higher diffusion rate of hydrogen in Pd electrodes at elevated temperatures enhanced interactions between hydrogen and oxygen, forming a highly conductive interfacial layer on the a-IGZO surface. The *R*_C_ slightly decreased to 5.9 Ω cm at a H_2_ annealing temperature of 200 °C, while Δ*L* significantly increased to 0.818 μm (Figure 2e), indicating that a higher annealing temperature causes a thicker highly conductive interfacial layer formation owing to the excessively rapid diffusion rate of hydrogen in both Pd and the a-IGZO.^43−45^ Thus, TLM results showed that the Pd-based hydrogen transfer pathway effectively transports hydrogen to the interface with a-IGZO within a hydrogen annealing atmosphere. Furthermore, *R*_C_ and Δ*L* variations with H_2_ annealing time are shown in Figure 2f for different temperatures. H_2_ annealing at 100 °C requires 20 min to achieve ohmic contact, which is attributed to the excessive hydrogen solubility of Pd with lower hydrogen desorption (Figure 1f) at the contact interface and the decreased efficiency of the hydrogen reaction with a-IGZO at 100 °C. Conversely, *R*_C_ decreased more rapidly at higher annealing temperatures. At 200 °C, the contact resistance can be reduced to below 10 Ω cm in 6 min, which is correlated with the higher diffusion rate of hydrogen in Pd at elevated temperatures. However, higher annealing temperatures also lead to a rapid reduction in the effective channel length, which may lead to a strong short-channel effect.^46^ Therefore, it turned out the 5% H_2_ atmosphere annealing at 150 °C in 10 min is the optimal condition for the a-IGZO TFTs with Pd electrodes.

### Characterization of the Pd–IGZO Interface

**Figure 3:**
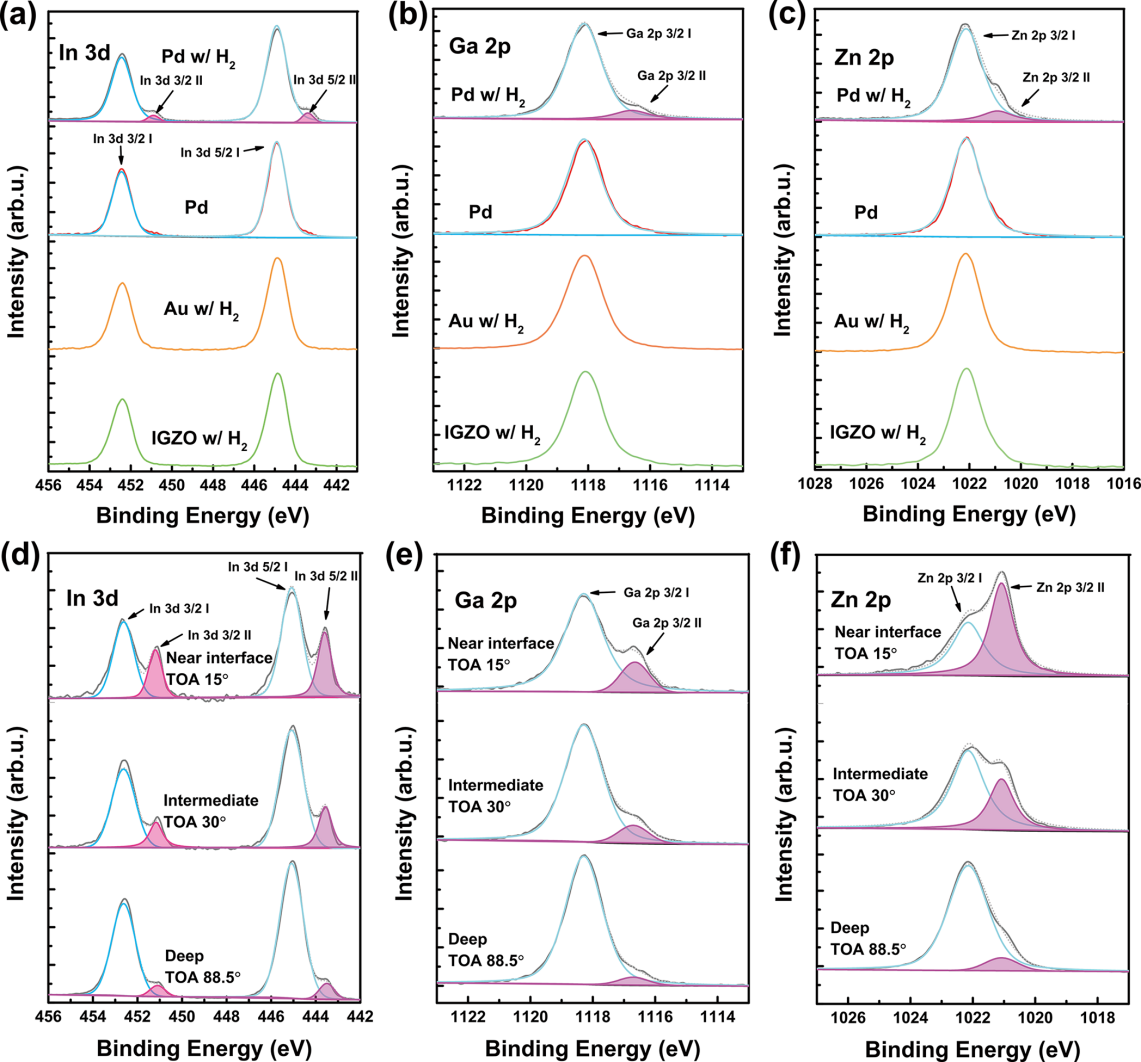
Chemical state of the a-IGZO thin-film. HAXPES spectra of (a) In 3d, (b) Ga 2p, and (c) Zn 2p core-levels for 5 nm thick Pd-passivated a-IGZO thin films with and without H_2_ annealing treatment (5% H_2_ atmosphere at 150 °C in 10 min), 5 nm thick Au-passivated a-IGZO thin film with H_2_ annealing treatment, and nonpassivated a-IGZO thin film with H_2_ annealing treatment, respectively, obtained at TOA = 88.5°. (d) In 3d, (e) Ga 2p, and (f) Zn 2p HAXPES spectra obtained at TOAs of 15° (near interface), 30° (intermediate), and 88.5° (deep) for Pd-passivated a-IGZO thin films with H_2_ annealing treatment.

To investigate the effect of postannealing in a 5% H_2_ atmosphere on the Pd–IGZO interface, a-IGZO thin films passivated with Pd (5 nm) or Au (5 nm) and without passivation were prepared for HAXPES measurements. Figure 3 shows the HAXPES spectra for the In 3d, Ga 2p, and Zn 2p core-levels of samples. To maximize the probe-depth (effective inelastic mean free path of photoelectrons: λ_eff_) in HAXPES, the takeoff angle of photoelectrons (TOA) was set to 88.5° with respect to the sample surfaces to detect the photoelectrons emitted from the deeply buried interface of a-IGZO film in Figure 3a–c. The Pd-passivated a-IGZO film exhibited distinct metallic peaks for In^0^ (In 3d 3/2 II and In 3d 5/2 II), Ga^0^ (Ga 2p 3/2 II), and Zn^0^ (Zn 2p 3/2 II) species after H_2_ annealing treatment (5% H_2_ atmosphere at 150 °C in 10 min), in contrast to the as-fabricated thin films. However, the a-IGZO thin films passivated with Au did not show metallic peaks after H_2_ annealing treatment, and the same spectra were observed for the a-IGZO thin films without a metal passivation layer. The appearance of metallic peaks is attributed to intense hydrogenation reactions occurring at the Pd and a-IGZO interface, which is often observed in strong metal–support interactions between Pd and oxides in catalysis.^47^ During H_2_ annealing treatment, H_2_ spontaneously dissociates into atomic hydrogen on the exposed Pd surface, and highly active atomic hydrogens are generated and diffused, followed by the reaction with oxide ions in the lattice, resulting in the formation of oxygen vacancies and the reduction of surface metal oxides.^48,49^ The neutral metal atom peaks indicate the formation of a high electron density and a highly conductive interfacial layer at the contact interface between a-IGZO and Pd, which reduces the contact resistance and eliminates the Schottky barrier. The absence of neutral atom peaks in the Au-passivated a-IGZO thin films after H_2_ annealing treatment indicates that no significant reduction reaction occurs at the a-IGZO–Au interface during postannealing, which is attributed to the low hydrogen diffusion rate of H_2_ in Au at low-temperature and less chemical reactivity of H_2_. The unpassivated bare a-IGZO thin films did not exhibit neutral metal peaks as well as Au-passivated samples after the H_2_ annealing treatment. Although the surface of a-IGZO was exposed to a hydrogen atmosphere during the annealing treatment, the reactivity of H_2_ molecules was much lower than that of the dissociated hydrogen atom,^50^ which was insufficient to react with a substantial amount of oxygen at low temperatures and within a short time to form a highly conductive layer. To clarify the depth distribution of the neutral metal peak in the buried a-IGZO film underneath the Pd passivation layer, TOA dependence of the In 3d, Ga 2p, and Zn 2p spectra was measured as shown in Figure 3d–f. By changing the TOA in HAXPES, in which λ_eff_ is proportional to sin(TOA), three different TOAs measurements at 15°, 30°, and 88.5° give the near-interface, intermediate, and deep a-IGZO sensitive depth information, respectively. The enhancement of peaks for the In^0^, Ga^0^, and Zn^0^ species in the case of TOA = 15°, as observed in Figure 3d–f, respectively, indicates that a more intense reduction reaction of hydrogen on a-IGZO would occur at a position closer to Pd. Notable enhancement for Zn^0^ compared to In^0^ and Ga^0^ is attributed to the facile reaction between Pd and ZnO during the annealing treatment, as evidenced by the X-ray diffraction (XRD) pattern (Figure S1). The peak shift of Pd 111 diffraction indicates the formation of intermetallic PdZn_*x*_ after the annealing process,^49,51,52^ which is also one of the reasons for the formation of the highly conductive interfacial layer. Hence, the formation of the highly conductive interfacial layer is attributed to the metallization resulting from hydrogen interacting with oxygen in a-IGZO at the interface, accompanied by the formation of an intermetallic phase between Pd and Zn, which eliminates the Schottky barrier and reduces the contact resistance between the metal and a-IGZO.

### TFT Performance and the Pd–IGZO Interface

**Figure 4:**
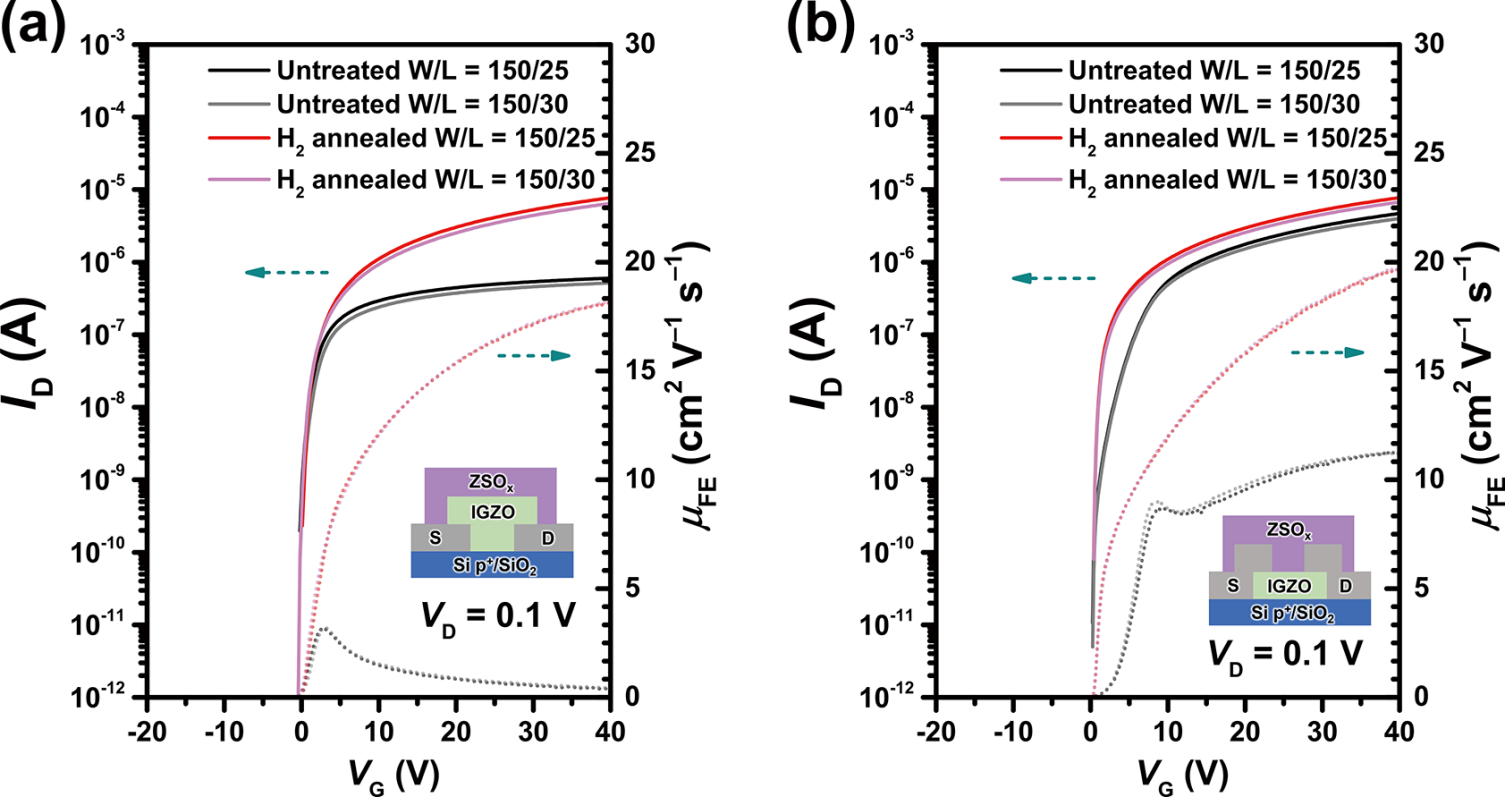
Electrical performance of the TFTs. The transfer characteristics (solid) and field effect mobility (dots) of a-IGZO TFTs with Pd electrodes passivated by 50 nm ZSO_*x*_ before and after H_2_ annealing treatment (150 °C for 10 min in a 5% H_2_ atmosphere), while the structures are (a) BGBC and (b) BGTC with *W*/*L* of 150/30 and 150/25 μm, respectively. (*V*_D_ = 0.1 V).

To demonstrate the application of the hydrogen transfer pathway based on Pd electrodes in a-IGZO TFTs, devices with two different structures, BGBC and bottom gate-top contact (BGTC), were fabricated as shown in Figure 4a,b, both covered with a 50-nm-thick ZSO_*x*_ passivation layer. To precisely determine the field-effect mobility, the transfer characteristics and mobility of TFTs with different width-to-length ratios (*W*/*L* = 150/30 and 150/25) were extracted. Compared with the top-contact structure (Figure 4b) of a-IGZO TFTs, the bottom-contact devices (Figure 4a) exhibited more pronounced contact issues before the H_2_ annealing treatment, causing a significantly lower drain current (*I*_D_) and a lower μ_FE_ of 3.2 cm^2^ V^–1^ s^–1^ (compared to that of the top-contact structure, 11.5 cm^2^ V^–1^ s^–1^). The higher contact resistance in the bottom-contact structure is tentatively attributed to the effect of the unfilled region and partial oxidation during a-IGZO sputtering at the interface between the channel and electrodes.^53,54^ Due to the uninterrupted supply of highly active hydrogen to the interface between the a-IGZO channel and electrodes facilitated by the Pd electrodes during 150 °C postannealing in a 5% H_2_ atmosphere for 10 min, the a-IGZO interface underwent metallization, reducing the contact resistance and total resistance ratio as shown in Figure S2 and significantly enhancing the drain current of the a-IGZO TFTs after H_2_ annealing treatment. For both the BGBC and BGTC structures, μ_FE_ increased to 20.0 cm^2^ V^–1^ s^–1^ after H_2_ annealing treatment. In contrast, the passivated a-IGZO TFTs with Au electrodes showed no change in μ_FE_ even after H_2_ annealing treatment (Figure S3), confirming the highly active hydrogen and the strong hydrogen transfer ability by utilizing Pd electrodes during H_2_ annealing treatment. Devices with varying *W*/*L* demonstrated consistent mobility results, indicating that the influence of minimal Δ*L* on effective mobility was negligible, thereby ruling out the possibility of short-channel effects. Figure S4a,b shows the statistical device parameters with small variations in μ_FE_ and *V*_T_ indicating the uniformity and reliability of the processes. The electrical performance of the sputtered a-IGZO TFTs presented in this work is excellent compared with those reported in recent studies, as listed in Table S1. Therefore, ZSO_*x*_-passivated a-IGZO TFTs demonstrate the high potential of Pd-based hydrogen transfer pathways for future applications of IGZO-based memory and display devices with complex architectures.

**Figure 5:**
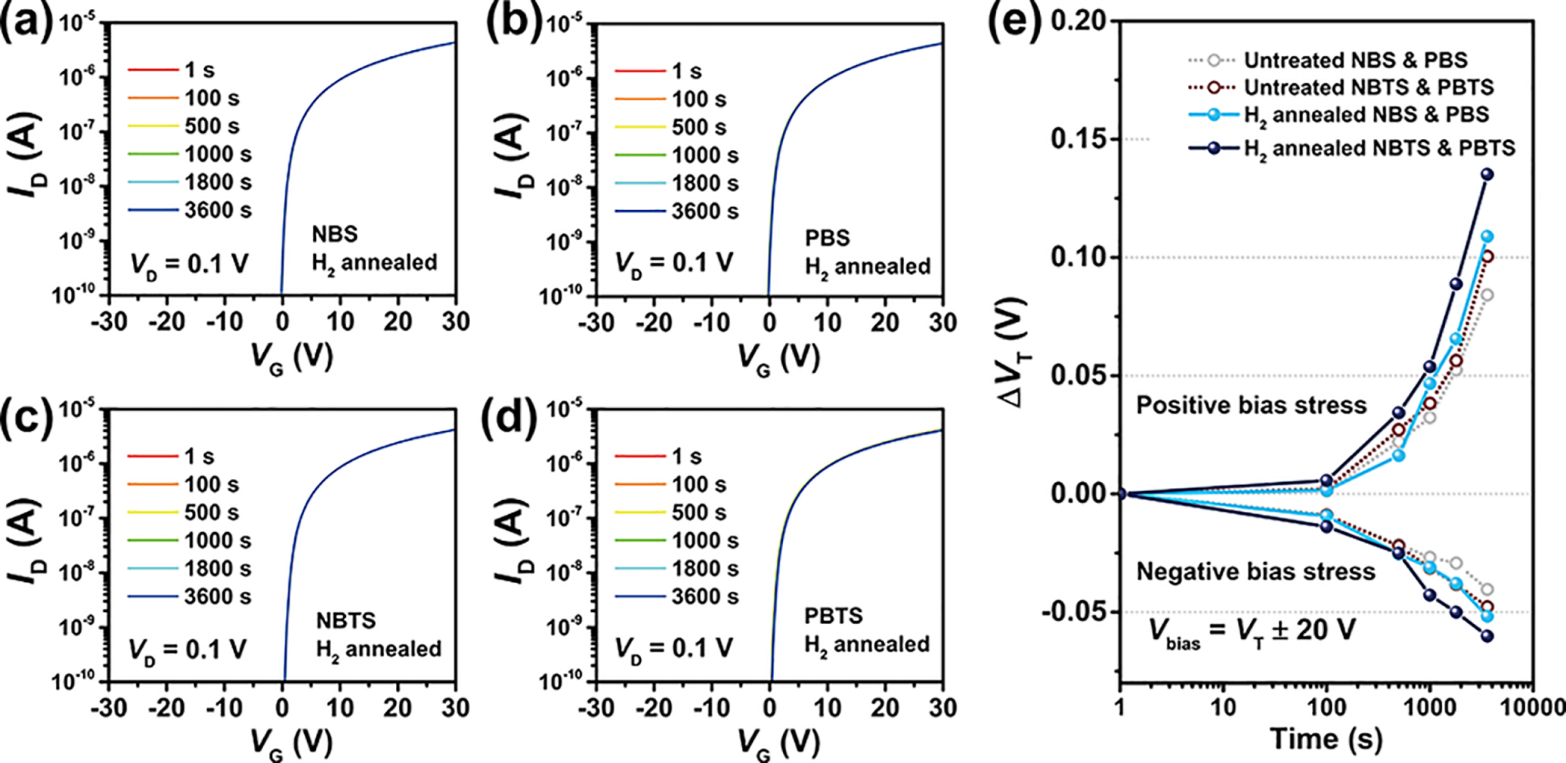
Bias stability of the TFTs. Transfer characteristics of H_2_-annealed (5% H_2_ atmosphere at 150 °C in 10 min) a-IGZO TFTs (BGBC) with Pd electrodes passivated by ZSO_*x*_ under (a) NBS, (b) PBS, (c) NBTS (60 °C), and (d) PBTS (60 °C) for 1 h (*V*_bias_ = *V*_T_ ± 20 V). (e) Comparison of the threshold voltage shift of untreated a-IGZO TFTs under NBS, PBS, NBTS, and PBTS with a-IGZO TFTs after H_2_ annealing treatment under NBS, PBS, NBTS, and PBTS for 1 h.

Protons in amorphous oxides are prone to drift within the channel under the electric field, thereby affecting the bias stability of amorphous oxide TFTs under long-time bias gate voltage.^55,56^ To examine the effect of H_2_ annealing treatment on the bias stability of a-IGZO TFTs with bottom contact Pd electrodes, gate biases (*V*_bias_) of *V*_T_ ± 20 V were applied to the TFTs for 1 h, and the threshold voltage drift (Δ*V*_T_) results were obtained as shown in Figure 5. As shown in Figure 5a, after application of an NBS for 1 h, the *V*_T_ drifted by only −0.05 V (Figure 5e). Alternatively, applying a PBS for 1 h showed a *V*_T_ drift of only +0.11 V, which is slightly larger than those of devices without H_2_ annealing treatment but still at an excellent level of bias stability. At high temperatures, the migration of hydrogen atoms in the amorphous oxide becomes more pronounced,^55^ making high-temperature bias stress testing a more sensitive indicator of the level of mobile hydrogen atoms in the channel. As shown in Figure 5c,d, the results of negative gate bias temperature stress (NBTS) and positive gate bias temperature stress (PBTS) indicate that the bias stress tests at 60 °C did not result in significant threshold voltage shifts, with corresponding values of −0.06 and +0.14 V, respectively. These results indicate that H_2_ annealing treatment with a Pd electrode has a negligible influence on the bias stability of ZSO_*x*_-passivated a-IGZO TFTs. The excellent bias stability at high temperatures and low hysteresis effect (Figure S4c) may be ascribed to the minimal mobile hydrogen ion content preserved within the a-IGZO channel after H_2_ annealing treatment, which indicates a lower effect of hydrogen diffused into the channel during the annealing treatment. At the same time, it reaffirms the protective role of the ZSO_*x*_ passivation layer, safeguarding the a-IGZO back channel from the influence of hydrogen and other impurities.^41^ The exceptional bias stability ensures minimal side effects of H_2_ annealing treatment on a-IGZO devices employing Pd electrodes, ensuring bias stability for future applications.

**Figure 6:**
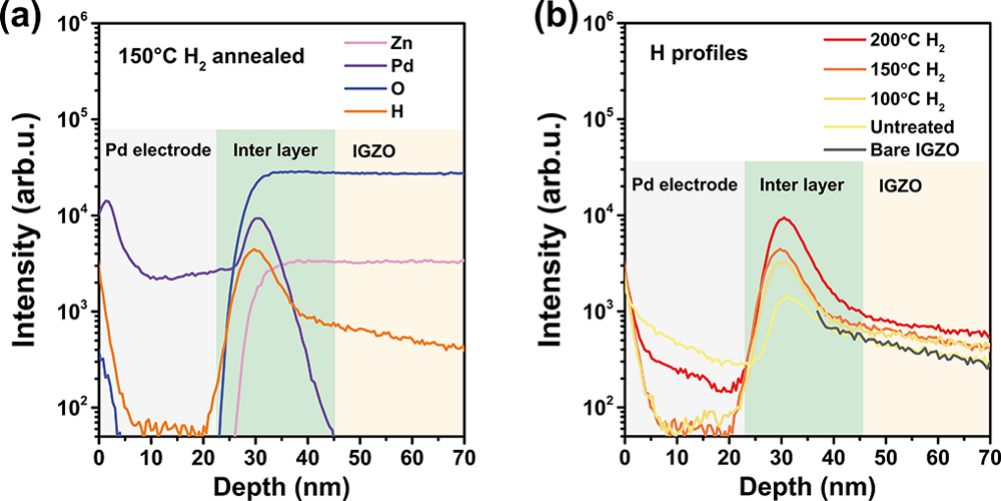
SIMS depth profile. (a) Depth profiles of H, Zn, Pd, and O for Pd-passivated a-IGZO thin film with H_2_ annealing treatment at 150 °C in 10 min. (b) H depth profiles for bare a-IGZO thin films (note that the top surface of bare a-IGZO is located at 36 nm) and Pd-passivated a-IGZO thin films before and after H_2_ annealing treatment at 100, 150, and 200 °C in 10 min.

To further explore the influence of H_2_ annealing treatment on the bias stability of TFT devices, depth profiles obtained by SIMS directly reveal the distribution of hydrogen at the Pd/a-IGZO interface. As shown in Figure 6a, 150 °C H_2_ annealing treatment leads to the formation of an interface layer at the junction of Pd and a-IGZO, characterized by a significantly high hydrogen content. The result also proves the efficient diffusion of hydrogen from the atmosphere to the electrode–oxide semiconductor interface at relatively low temperatures when Pd serves as the high-efficiency hydrogen transfer pathway. Moreover, as shown in Figure 6b, the intensity of hydrogen increased with the annealing temperature from 100 to 200 °C, which indicates the higher transfer efficiency of Pd at a high temperature, aligning with the trend in the diffusion rate of hydrogen in Pd (Figure 1e) and the contact resistance with the annealing temperature (Figure 2f). As for the presence of hydrogen in the interlayer position, it can be considered that after Pd transferred atomic H^0^ to the electrode–oxide semiconductor interface, H^0^ interacts with the interface oxide, converting into protonic hydrogen, which then reacts with oxygen at the interface IGZO to form OH groups, accumulating at the interface. In the a-IGZO layer, only a slight increase in intensity was observed compared to the untreated sample and bare a-IGZO sample, as shown in Figure 6b. This observation suggests a low H content within the channel, indicating a minimal influence on device stability during bias testing, which is consistent with prior bias stability results. On the other hand, no residual hydrogen was detected in the Pd layer after H_2_ annealing treatment, so there is no concern about the occurrence of secondary effects due to residual excess hydrogen diffusion into a-IGZO during high-temperature TFT operations. Thus, Pd, as a hydrogen transport metal electrode, efficiently transports a substantial amount of hydrogen to the electrode–oxide semiconductor interface during low-temperature H_2_ annealing treatment with less diffusion into the a-IGZO channel layer, and then the accumulated hydrogen induces the metallization of the a-IGZO interface.

## Conclusions

In this study, an approach utilizing Pd electrodes as hydrogen transfer pathways to address the issue of internal contact interface contact resistance in oxide semiconductor devices was proposed. The postannealing in a hydrogen atmosphere enables hydrogen to rapidly reach the oxide–Pd interface in the device interior up to a depth of 100 μm via the Pd electrodes. This method utilizes the exceptionally high diffusion rate of hydrogen in Pd at relatively low temperatures as well as its suitable solubility. When the charge transition level of *E*(H^+^/H^–^) is higher than the *E*_CBM_ of oxide semiconductors, diffused atomic hydrogen induces a metallization reaction at the contact interface with the oxide semiconductor, thereby generating carrier electrons and forming a highly conductive interlayer. According to Figure 1c, high-mobility oxide semiconductor materials with high (In + Sn) fraction correspond to this case.^41^ Thus, this method can be applied to high-mobility oxide TFTs. It turned out 150 °C was the optimal temperature under annealing in a 5% H_2_ atmosphere at various temperatures for 10 min. The TLM measurement was employed to assess the contact resistance between the a-IGZO and the Pd electrodes. The results indicated a transition from Schottky to ohmic contact after annealing treatment at 150 °C in a H_2_ atmosphere for 10 min, and higher temperatures with long annealing time resulted in a significant reduction in the effective channel length. Further analysis of the a-IGZO and Pd interfaces using HAXPES and SIMS revealed that the highly active hydrogen in the Pd electrodes during H_2_ annealing treatment induced the metallization of the a-IGZO surface and the formation of the intermetallic PdZn_*x*_, constructing a high-electron-density interface layer and thereby reducing the contact resistance. The a-IGZO TFTs with Pd electrodes exhibited a substantial enhancement in drain current and μ_FE_ after H_2_ annealing treatment, with a promoted μ_FE_ approaching 20 cm^2^ V^–1^ s^–1^. Meanwhile, the excellent bias stability of a-IGZO TFTs indicated a negligible effect of hydrogen diffused in the channel after H_2_ annealing treatment with Pd electrodes. This approach for low-contact resistance formation between the AOS and the metal, specifically tailored for complex device architectures, provides a highly valuable solution for future applications of AOSs in memory and display devices.

## Methods

### Device Fabrication

To fabricate a-IGZO TFTs for TLM and transfer characteristics measurement, heavily doped p-type Si wafers were used as bottom-gate substrates with a 150 nm thermally oxidized SiO_2_ gate insulator. For the BGBC structure, a 5 nm Ti layer was sputtered for better adhesion of metal electrodes on SiO_2_, and 30 nm Pd or Au was thermally evaporated as the source and drain (S/D) electrodes. Subsequently, the 20 nm a-IGZO (In:Ga:Zn = 1:1:1 atom %) channel was deposited by 150 W RF magnetron sputtering under chamber base pressure of 0.4 Pa, and an O_2_ partial pressure of 0.25%. Following channel deposition, a larger area of 50 nm ZSO_*x*_ passivation layer, with a Zn:Si ratio of 75:25 atom %, was deposited under 0.4 Pa with an O_2_ partial pressure of 25%. For the BGTC structure, 30 nm Pd electrodes without the Ti layer were thermally evaporated following the sputtering of a 20 nm a-IGZO channel layer for comparison, and then a 50 nm ZSO_*x*_ passivation layer was sputtered under the same conditions. To complete the fabrication, all passivated devices were annealed at 300 °C in an ambient atmosphere for 1 h. The electrodes, channel layer, and passivation layer were all patterned by photolithography. Channel length (*L*) from 10 to 160 μm and a channel width (*W*) of 500 μm were patterned for TLM measurement, and an *L* of 25 or 30 μm and a *W* of 150 μm were patterned for TFT transfer characteristics measurement. For the H_2_ annealing treatment, fabricated devices were postannealed in a 5% hydrogen atmosphere at 1 atm. in 10 min, and the annealing temperatures of 100, 150, and 200 °C were used for comparison.

### Characterizations

The electrical characterizations of the TFTs were carried out under vacuum using the Keysight B2912A measurement unit. The contact resistance of the devices was extracted by using TLM.^42^ The thickness of the thin films was measured by X-ray reflectometry (Smart Lab, Rigaku). HAXPES measurements at room temperature were performed at the BL09XU undulator beamline (photon energy = 5.95 keV) in SPring-8. The binding energy was referred to the Fermi level of an Au plate, and the overall energy resolution was set to ∼210 meV. The *E*-vector of the photon was set to parallel to the direction of photoelectrons detected by a high-resolution electron analyzer (Scienta Omicron R4000). The components of the passivated a-IGZO layers were measured by SIMS (ION TOF TOF-SIMS^5^). The Pd-covered a-IGZO thin films were clarified by XRD (Smart Lab, Rigaku) via the 2-θ method. The electrical properties of a-IGZO and ZSO_*x*_-passivated a-IGZO were evaluated by ac-field Hall effect measurements (ResiTest8400, TOYO).

## Supporting Information Available

Hall measurement results; XRD spectra; ratio of contact resistance and total resistance of a-IGZO channel; transfer characteristics of a-IGZO TFTs with Au; statistical data of TFTs; comparison of mobility and contact resistance of a variety of IGZO TFTs (PDF)

## References

[ref1] Nomura K.; Ohta H.; Takagi A.; Kamiya T.; Hirano M.; Hosono H. Room-temperature fabrication of transparent flexible thin-film transistors using amorphous oxide semiconductors. Nature 2004, 432 (7016), 488–492. 10.1038/nature03090. 15565150

[ref2] Kamiya T.; Hosono H. Material characteristics and applications of transparent amorphous oxide semiconductors. NPG Asia Mater. 2010, 2 (1), 15–22. 10.1038/asiamat.2010.5.

[ref3] Ide K.; Nomura K.; Hosono H.; Kamiya T. Electronic Defects in Amorphous Oxide Semiconductors: A Review. Phys. Status Solidi A 2019, 216 (5), 1800372 10.1002/pssa.201800372.

[ref4] Hosono H., Kumomi H., Ed. Amorphous Oxide Semiconductors; Wiley, 2022.

[ref5] Belmonte A.; Oh H.; Rassoul N.; Donadio G. L.; Mitard J.; Dekkers H.; Delhougne R.; Subhechha S.; Chasin A.; Setten M. J. v.; Kljucar L.; Mao M.; Puliyalil H.; Pak M.; Teugels L.; Tsvetanova D.; Banerjee K.; Souriau L.; Tokei Z.; Goux L.; Kar G. S. Capacitor-less, Long-Retention (>400s) DRAM Cell Paving the Way towards Low-Power and High-Density Monolithic 3D DRAM. Proceedings, 2020 IEEE International Electron Devices Meeting (IEDM), Dec 12–18, 2020; IEEE, 2020; pp 28.2.1–28.2.4.

[ref6] Yan G.; Yang H.; Liu W.; Zhou N.; Hu Y.; Shi Y.; Gao J.; Tian G.; Zhang Y.; Fan L.; Wang G.; Xu G.; Bi J.; Yin H.; Zhao C.; Luo J. Mechanism Analysis of Ultralow Leakage and Abnormal Instability in InGaZnO Thin-Film Transistor Toward DRAM. IEEE Trans. Electron Devices 2022, 69 (5), 2417–2422. 10.1109/TED.2022.3159266.

[ref7] Tang W.; Liu J.; Sun C.; Zheng Z.; Liu Y.; Yang H.; Jiang C.; Ni K.; Gong X.; Li X. Low-Power and Scalable BEOL-Compatible IGZO TFT eDRAM-Based Charge-Domain Computing. IEEE Trans. Circuits Syst. I-Regul. Pap. 2023, 70 (12), 5166–5179. 10.1109/TCSI.2023.3317170.

[ref8] Hu Q.; Gu C.; Li Q.; Zhu S.; Liu S.; Li Y.; Zhang L.; Huang R.; Wu Y. True Nonvolatile High-Speed DRAM Cells Using Tailored Ultrathin IGZO. Adv. Mater. 2023, 35 (20), 2210554 10.1002/adma.202210554. 36892994

[ref9] Kim J.; Hong A. J.; Kim S. M.; Song E. B.; Park J. H.; Han J.; Choi S.; Jang D.; Moon J.-T.; Wang K. L. Novel Vertical-Stacked-Array-Transistor (VSAT) for ultra-high-density and cost-effective NAND flash memory devices and SSD (solid state drive). Proceedings, 2009 Symposium on VLSI Technology, June 15–17, 2009; IEEE, 2009; pp 186–187.

[ref10] Park K. T.; Nam S.; Kim D.; Kwak P.; Lee D.; Choi Y. H.; Choi M. H.; Kwak D. H.; Kim D. H.; Kim M. S.; Park H. W.; Shim S. W.; Kang K. M.; Park S. W.; Lee K.; Yoon H. J.; Ko K.; Shim D. K.; Ahn Y. L.; Ryu J.; Kim D.; Yun K.; Kwon J.; Shin S.; Byeon D. S.; Choi K.; Han J. M.; Kyung K. H.; Choi J. H.; Kim K. Three-Dimensional 128 Gb MLC Vertical nand Flash Memory With 24-WL Stacked Layers and 50 MB/s High-Speed Programming. IEEE J. Solid-State Circuits 2015, 50 (1), 204–213. 10.1109/JSSC.2014.2352293.

[ref11] Choi S.; Kim B.; Jeong J. K.; Song Y. H. A Novel Structure for Improving Erase Performance of Vertical Channel NAND Flash With an Indium-Gallium-Zinc-Oxide Channel. IEEE Trans. Electron Devices 2019, 66 (11), 4739–4744. 10.1109/TED.2019.2942935.

[ref12] Ahn H.-M.; Kwon Y.-H.; Seong N.-J.; Choi K.-J.; Hwang C.-S.; Yoon S.-M. Impact of Strategic Approaches for Improving the Device Performance of Mesa-shaped Nanoscale Vertical-Channel Thin-Film Transistors Using Atomic-Layer Deposited In-Ga-Zn-O Channel Layers. Electron. Mater. Lett. 2022, 18 (3), 294–303. 10.1007/s13391-022-00336-w.

[ref13] Bae S. H.; Ryoo H. J.; Yang J. H.; Kim Y. H.; Hwang C. S.; Yoon S. M. Influence of Reduction in Effective Channel Length on Device Operations of In-Ga-Zn-O Thin-Film Transistors With Variations in Channel Compositions. IEEE Trans. Electron Devices 2021, 68 (12), 6159–6165. 10.1109/TED.2021.3117188.

[ref14] Bae S.-H.; Yang J.-H.; Kim Y.-H.; Kwon Y. H.; Seong N.-J.; Choi K.-J.; Hwang C.-S.; Yoon S.-M. Roles of Oxygen Interstitial Defects in Atomic-Layer Deposited InGaZnO Thin Films with Controlling the Cationic Compositions and Gate-Stack Processes for the Devices with Subμm Channel Lengths. ACS Appl. Mater. Interfaces 2022, 14 (27), 31010–31023. 10.1021/acsami.2c07258. 35785988

[ref15] Rivas-Aguilar M. E.; Hernandez-Como N.; Gutierrez-Heredia G.; Sánchez-Martínez A.; Ramirez M. M.; Mejia I.; Quevedo-López M. A. Specific contact resistance of IGZO thin film transistors with metallic and transparent conductive oxides electrodes and XPS study of the contact/semiconductor interfaces. Curr. Appl. Phys. 2018, 18 (7), 834–842. 10.1016/j.cap.2018.04.002.

[ref16] Shiah Y. S.; Sim K.; Ueda S.; Kim J.; Hosono H. Unintended Carbon-Related Impurity and Negative Bias Instability in High-Mobility Oxide TFTs. IEEE Electron Device Lett. 2021, 42 (9), 1319–1322. 10.1109/LED.2021.3101654.

[ref17] Choi S. H. High-Performance Oxide TFTs With Co-Sputtered Indium Tin Oxide and Indium-Gallium-Zinc Oxide at Source and Drain Contacts. IEEE Electron Device Lett. 2021, 42 (2), 168–171. 10.1109/LED.2020.3047389.

[ref18] Park J.; Shin M.; Yi J. Comparative study of aluminum and nickel contact electrodes for indium-tin-zinc oxide thin film transistors using oxygen vacancy diffusion model. Mater. Sci. Semicond. Process. 2020, 120, 105253 10.1016/j.mssp.2020.105253.

[ref19] Lee D.-H.; Kwon Y.-H.; Seong N.-J.; Choi K.-J.; Kim G.; Yoon S.-M. Analysis on Contact Resistance and Effective Channel Length of Thin Film Transistors Using Composition-Modified In-Ga-Zn-O Active Channels Prepared with Atomic Layer Deposition and Various Electrode Materials. ACS Appl. Electron. Mater. 2022, 4 (12), 6215–6228. 10.1021/acsaelm.2c01342.

[ref20] Shimura Y.; Nomura K.; Yanagi H.; Kamiya T.; Hirano M.; Hosono H. Specific contact resistances between amorphous oxide semiconductor In-Ga-Zn-O and metallic electrodes. Thin Solid Films 2008, 516 (17), 5899–5902. 10.1016/j.tsf.2007.10.051.

[ref21] Shi Y.; Shiah Y.-S.; Sim K.; Sasase M.; Kim J.; Hosono H. High-performance a-ITZO TFTs with high bias stability enabled by self-aligned passivation using a-GaOx. Appl. Phys. Lett. 2022, 121 (21), 212101 10.1063/5.0123253.

[ref22] Peng H.; Chang B.; Fu H.; Yang H.; Zhang Y.; Zhou X.; Lu L.; Zhang S. Top-Gate Amorphous Indium-Gallium-Zinc-OxideThin-Film Transistors With Magnesium Metallized Source/Drain Regions. IEEE Trans. Electron Devices 2020, 67 (4), 1619–1624. 10.1109/TED.2020.2975211.

[ref23] Ye Z.; Lu L.; Wong M. Zinc-Oxide Thin-Film Transistor With Self-Aligned Source/Drain Regions Doped With Implanted Boron for Enhanced Thermal Stability. IEEE Trans. Electron Devices 2012, 59 (2), 393–399. 10.1109/TED.2011.2175398.

[ref24] Du Ahn B.; Shin H. S.; Kim H. J.; Park J.-S.; Jeong J. K. Comparison of the effects of Ar and H2 plasmas on the performance of homojunctioned amorphous indium gallium zinc oxide thin film transistors. Appl. Phys. Lett. 2008, 93 (20), 203506 10.1063/1.3028340.

[ref25] Park H.; Yun J.; Park S.; Ahn I.-s.; Shin G.; Seong S.; Song H.-J.; Chung Y. Enhancing the Contact between a-IGZO and Metal by Hydrogen Plasma Treatment for a High-Speed Varactor (>30 GHz). ACS Appl. Electron. Mater. 2022, 4 (4), 1769–1775. 10.1021/acsaelm.2c00028.

[ref26] Jang H.; Lee S. J.; Porte Y.; Myoung J.-M. Selective metallization of amorphous-indium-gallium-zinc-oxide thin-film transistor by using helium plasma treatment. Semicond. Sci. Technol. 2018, 33 (3), 035011 10.1088/1361-6641/aaa9e7.

[ref27] Van de Walle C. G.; Neugebauer J. Universal alignment of hydrogen levels in semiconductors, insulators and solutions. Nature 2003, 423 (6940), 626–628. 10.1038/nature01665. 12789334

[ref28] Hosono H. Transparent Oxide Semiconductors: Fundamentals and Recent Progress. In Transparent Electronics; Facchetti A., Marks T. J., Eds.; Wiley, 2010; pp 31–59.

[ref29] Hanyu Y.; Domen K.; Nomura K.; Hiramatsu H.; Kumomi H.; Hosono H.; Kamiya T. Hydrogen passivation of electron trap in amorphous In-Ga-Zn-O thin-film transistors. Appl. Phys. Lett. 2013, 103 (20), 202114 10.1063/1.4832076.

[ref30] Bang J.; Matsuishi S.; Hosono H. Hydrogen anion and subgap states in amorphous In-Ga-Zn-O thin films for TFT applications. Appl. Phys. Lett. 2017, 110 (23), 232105 10.1063/1.4985627.

[ref31] Bergh A. A. Atomic hydrogen as a reducing agent. Bell Syst. Tech. J. 1965, 44 (2), 261–271. 10.1002/j.1538-7305.1965.tb01661.x.

[ref32] Katsuta H.; Farraro R. J.; McLellan R. B. The diffusivity of Hydrogen in palladium. Acta Metall. 1979, 27 (7), 1111–1114. 10.1016/0001-6160(79)90128-7.

[ref33] Scully J. R.; Young G. A. Jr; Smith S. W. Hydrogen Solubility, Diffusion and Trapping in High Purity Aluminum and Selected Al-Base Alloys. Mater. Sci. Forum 2000, 331–337, 1583–1600. 10.4028/www.scientific.net/MSF.331-337.1583.

[ref34] Ishikawa T.; McLellan R. B. The diffusivity of hydrogen in the noble metals at low temperature. Acta Metall. 1985, 33 (11), 1979–1985. 10.1016/0001-6160(85)90120-8.

[ref35] Ishikawa T.; McLellan R. B. The diffusivity of hydrogen in copper at low temperatures. J. Phys. Chem. Solids 1985, 46 (4), 445–447. 10.1016/0022-3697(85)90110-6.

[ref36] Miyoshi T.; Naito S.; Yamamoto M.; Doi M.; Kimura M. Diffusion of hydrogen in titanium, Ti88Al12 and Ti3Al. J. Chem. Soc., Faraday Trans. 1996, 92 (3), 483–486. 10.1039/ft9969200483.

[ref37] Wipf H. Introduction. In Hydrogen in Metals III: Properties and Applications; Wipf H., Ed.; Springer: Berlin, 1997; pp 1–4.

[ref38] Huang Y. C.; Fujita K.; Uchida H. Metal-Hydrogen System Phase Diagram. Materia Jpn. 1979, 18 (10), 694–703. 10.2320/materia1962.18.694.

[ref39] Yi L.; You-ping C.; Han S.; Gang Z. Hydrogen gas sensor based on palladium and yttrium alloy ultrathin film. Rev. Sci. Instrum. 2012, 83 (12), 125003 10.1063/1.4770329. 23278019

[ref40] Dekura S.; Kobayashi H.; Kusada K.; Kitagawa H. Hydrogen in Palladium and Storage Properties of Related Nanomaterials: Size, Shape, Alloying, and Metal-Organic Framework Coating Effects. ChemPhysChem 2019, 20 (10), 1158–1176. 10.1002/cphc.201900109. 30887646

[ref41] Shiah Y.-S.; Sim K.; Shi Y.; Abe K.; Ueda S.; Sasase M.; Kim J.; Hosono H. Mobility-stability trade-off in oxide thin-film transistors. Nat. Electron. 2021, 4 (11), 800–807. 10.1038/s41928-021-00671-0.

[ref42] Xu Y.; Liu C.; Amegadze P. S. K.; Park W.-T.; Long D. X.; Minari T.; Balestra F.; Ghibaudo G.; Noh Y.-Y. Significant roles of low-temperature post-metallization annealing in solution-processed oxide thin-film transistors. Appl. Phys. Lett. 2014, 105 (13), 133505 10.1063/1.4897003.

[ref43] Turnbull A. Hydrogen diffusion and trapping in metals. In Gaseous Hydrogen Embrittlement of Materials in Energy Technologies; Gangloff R. P., Somerday B. P., Eds.; Woodhead Publishing, 2012; pp 89–128.

[ref44] Noh H. Y.; Lee W.-G.; G. R. H.; Cha J.-H.; Kim J.-S.; Yun W. S.; Lee M.-J.; Lee H.-J. Hydrogen diffusion and its electrical properties variation as a function of the IGZO stacking structure. Sci. Rep. 2022, 12 (1), 19816 10.1038/s41598-022-24212-7. 36396967 PMC9672038

[ref45] Kim G. Evaluation of oxygen-vacancy concentration through simulated hydrogen diffusion in amorphous In-Ga-Zn-O. Comput. Mater. Sci. 2022, 203, 111109 10.1016/j.commatsci.2021.111109.

[ref46] Yang C. I.; Chang T. C.; Liao P. Y.; Chen L. H.; Chen B. W.; Chou W. C.; Chen G. F.; Lin S. C.; Yeh C. Y.; Tsai C. M.; Yu M. C.; Zhang S. Drain-Induced-Barrier-Lowing-Like Effect Induced by Oxygen-Vacancy in Scaling-Down via-Contact Type Amorphous InGaZnO Thin-Film Transistors. IEEE J. Electron Devices Soc. 2018, 6, 685–690. 10.1109/JEDS.2018.2837682.

[ref47] Wu P.; Tan S.; Moon J.; Yan Z.; Fung V.; Li N.; Yang S.-Z.; Cheng Y.; Abney C. W.; Wu Z.; Savara A.; Momen A. M.; Jiang D.-e.; Su D.; Li H.; Zhu W.; Dai S.; Zhu H. Harnessing strong metal-support interactions via a reverse route. Nat. Commun. 2020, 11 (1), 3042 10.1038/s41467-020-16674-y. 32546680 PMC7297808

[ref48] Rui N.; Wang Z.; Sun K.; Ye J.; Ge Q.; Liu C.-j. CO2 hydrogenation to methanol over Pd/In2O3: effects of Pd and oxygen vacancy. Appl. Catal., B 2017, 218, 488–497. 10.1016/j.apcatb.2017.06.069.

[ref49] Niu Y.; Liu X.; Wang Y.; Zhou S.; Lv Z.; Zhang L.; Shi W.; Li Y.; Zhang W.; Su D. S.; Zhang B. Visualizing Formation of Intermetallic PdZn in a Palladium/Zinc Oxide Catalyst: Interfacial Fertilization by PdHx. Angew. Chem., Int. Ed. 2019, 58 (13), 4232–4237. 10.1002/anie.201812292. 30650222

[ref50] Aireddy D. R.; Ding K. Heterolytic Dissociation of H2 in Heterogeneous Catalysis. ACS Catal. 2022, 12 (8), 4707–4723. 10.1021/acscatal.2c00584.

[ref51] Kim J.-H.; Mirzaei A.; Kim H. W.; Kim S. S. Pd functionalization on ZnO nanowires for enhanced sensitivity and selectivity to hydrogen gas. Sens. Actuators, B 2019, 297, 126693 10.1016/j.snb.2019.126693.

[ref52] Milligan C. A.; Seemakurthi R. R.; Gao J.; Greeley J. P.; Miller J. T.; Ribeiro F. H.; Zemlyanov D. Y. Structure-Controlled Chemical Properties of PdZn Near-Surface Alloys. J. Phys. Chem. C 2022, 126 (32), 13660–13674. 10.1021/acs.jpcc.2c01637.

[ref53] Gupta D.; Katiyar M.; Gupta D. An analysis of the difference in behavior of top and bottom contact organic thin film transistors using device simulation. Org. Electron. 2009, 10 (5), 775–784. 10.1016/j.orgel.2009.03.012.

[ref54] Nag M.; Bhoolokam A.; Steudel S.; Chasin A.; Groeseneken G.; Heremans P. Comparative study of source-drain contact metals for amorphous InGaZnO thin-film transistors. J. Soc. Inf. Dispersion 2014, 22 (6), 310–315. 10.1002/jsid.250.

[ref55] Jang J. T.; Ko D.; Choi S. J.; Kim D. M.; Kim D. H. Observation of Hydrogen-Related Defect in Subgap Density of States and Its Effects Under Positive Bias Stress in Amorphous InGaZnO TFT. IEEE Electron Device Lett. 2021, 42 (5), 708–711. 10.1109/LED.2021.3066624.

[ref56] Domen K.; Miyase T.; Abe K.; Hosono H.; Kamiya T. Positive Gate Bias Instability Induced by Diffusion of Neutral Hydrogen in Amorphous In-Ga-Zn-O Thin-Film Transistor. IEEE Electron Device Lett. 2014, 35 (8), 832–834. 10.1109/LED.2014.2327234.

