# Supplementary material for: Approach to Low Contact Resistance Formation on Buried Interface in Oxide Thin-Film Transistors: Utilization of Palladium-Mediated Hydrogen Pathway
Source: ACS Nano. 2024 Mar 22;18(13):9736–45. doi: 10.1021/acsnano.4c02101 (PMC10993643; doi:10.1021/acsnano.4c02101)
Supplement: Supplementary file 1 — nn4c02101_si_001.pdf [file nn4c02101_si_001.pdf]

Supporting Information

Approach to Low Contact Resistance Formation on Buried Interface in Oxide Thin-Film Transistors: Utilization of Palladium-Mediated Hydrogen Pathway

Yuhao Shi[1], *Masatake Tsuji*[1]*, *Hanjun Cho*[1], *Shigenori Ueda*[2], *Junghwan Kim*[1, 3]*, *and Hideo Hosono*[1, 4]*

[1]MDX Research Center for Element Strategy, International Research Frontiers Initiative, Tokyo Institute of Technology, Yokohama, 226-8503, Japan.

E-mail: ma-tsuji@mces.titech.ac.jp; hosono@mces.titech.ac.jp

[2]Research Center for Electronic and Optical Materials, National Institute for Materials Science (NIMS), Tsukuba, Ibaraki, 305-0044, Japan

[3]Graduate School of Semiconductor Materials and Devices Engineering, Ulsan National Institute of Science and Technology, Ulsan, 44919, Republic of Korea.

E-mail: j.kim@unist.ac.kr

[4]Research Center for Materials Nanoarchitectonics, NIMS, Tsukuba, Ibaraki, 305-0044, Japan

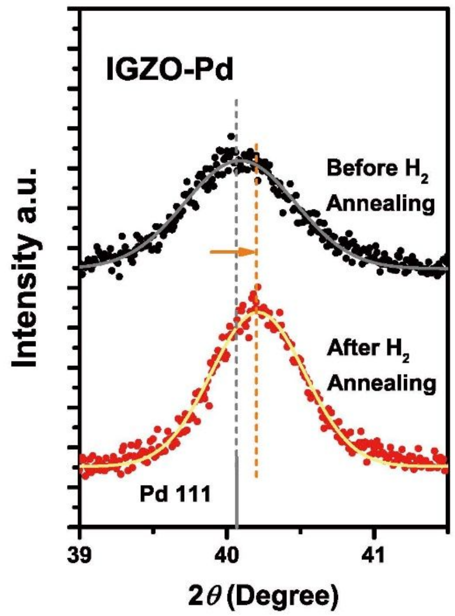

Figure S1. The XRD spectra of Pd-covered a-IGZO thin films before and after H_2 annealing treatment (150°C in 10 min at 5% H_2 atmosphere).

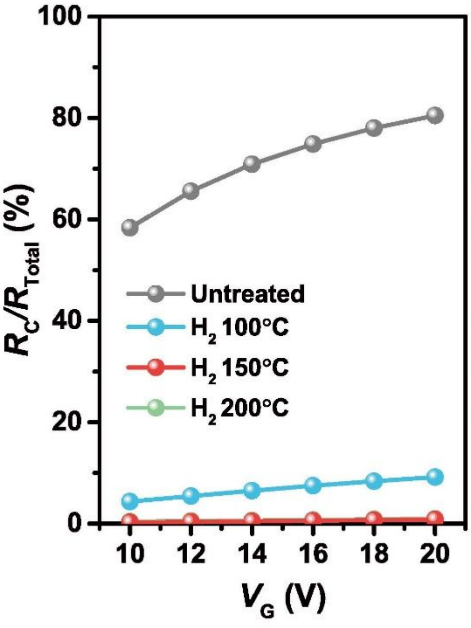

Figure S2. The ratio of contact resistance and total resistance of a-IGZO channel (*W*/*L* of 150/30 μm) with Pd electrodes before and after H_2 annealing treatment at 100°C, 150°C, and 200°C in 10 min (V_G = 10 – 20 V).

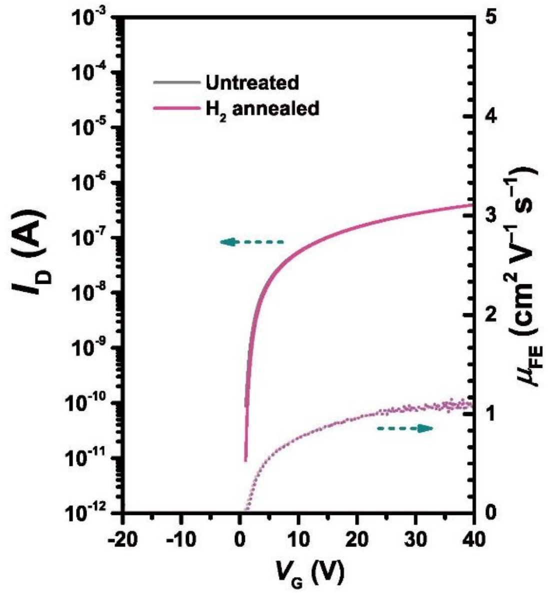

Figure S3. Transfer characteristics (solid) and linear mobility (dots) of a-IGZO thin-film transistors with Au electrodes passivated by 50 nm ZSO_x before and after H_2 annealing treatment (150°C in 10 min at 5% H_2 atmosphere).

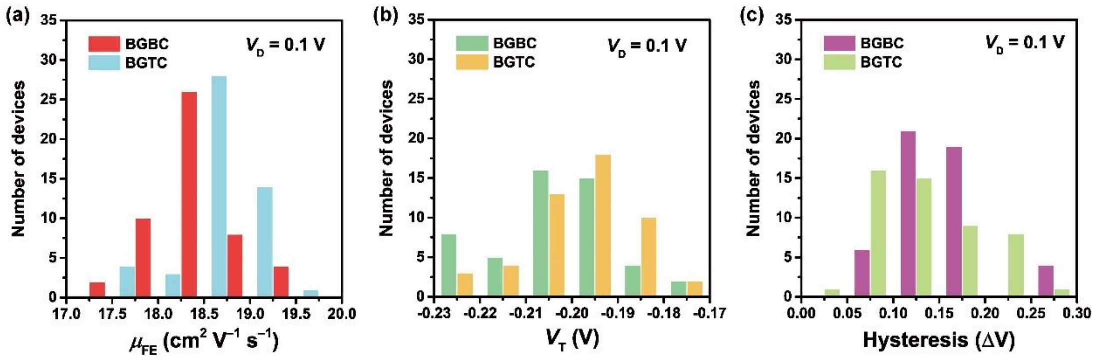

Figure S4. The statistical data of field-effect mobility, threshold voltage, and hysteresis effect of a-IGZO TFTs with Pd electrodes after H_2 annealing treatment for showing the uniformity and reliability of the processes, and the structures were BGBC and BGTC, respectively.

Table S1. Comparison of mobility and contact resistance of a variety of IGZO TFTs.

Channel	μ_{FE} ($cm^2\ V^{-1}\ s^{-1}$)	Contact resistance (Ω cm)	NBS (V)	PBS (V)	Electrode material	Treatment process	Structure	Reference
**Sputter a-IGZO**	**18.1**	**6.1**	**−0.05**	**0.11**	**Pd**	**H_2 annealing**	**BGBC**	**This work**
Sputter a-IGZO	13.2	~ 10^3	~ −0.5	~ 0.1	Ti/Au	Interlayer	BGBC	1
Sputter a-IGZO	14.1	~ 4×10^2	~ −0.5	~ 1.0	Ti/Au	Interlayer	BGTC	1
Sputter a-IGZO	8.8	~ 5×10^4	-	15.0	Al	He plasma	BGTC	2
Sputter a-IGZO	19.5	2.1	~ −0.1	~ 0.1	Mo	Mg-diffusion	TGTC	3
Sputter a-IGZO	7.3	75	-	-	IZO	H_2 plasma	BGTC	4
Sputter a-IGZO	7.5	0.9	~ −0.4	~ 0.7	Mo	Ar plasma	BGTC	5
PLD a-IGZO	7.6	~ 20	-	-	Ti	Thermal annealing	BGTC	6
ALD IGZO	11.5	-	~ −0.15	-	Mo	-	BGBC	7
ALD IGZO	12.9	~ 80	-	~ 0.9	Mo	-	BGBC	8

Reference

(1) Nag, M.; Bhoolokam, A.; Steudel, S.; Chasin, A.; Groeseneken, G.; Heremans, P. Comparative study of source–drain contact metals for amorphous InGaZnO thin-film transistors. *J. Soc. Inf. Disp.* **2014,** *22* (6), 310-315.

(2) Jang, H.; Lee, S. J.; Porte, Y.; Myoung, J.-M. Selective metallization of amorphous-

indium–gallium–zinc-oxide thin-film transistor by using helium plasma treatment. *Semicond. Sci. Technol.* **2018,** *33* (3), 035011.

(3) Peng, H.; Chang, B.; Fu, H.; Yang, H.; Zhang, Y.; Zhou, X.; Lu, L.; Zhang, S. Top-Gate Amorphous Indium-Gallium-Zinc-OxideThin-Film Transistors With Magnesium Metallized Source/Drain Regions. *IEEE Trans. Electron Devices* **2020,** *67* (4), 1619-1624.

(4) Du Ahn, B.; Shin, H. S.; Kim, H. J.; Park, J.-S.; Jeong, J. K. Comparison of the effects of Ar and H2 plasmas on the performance of homojunctioned amorphous indium gallium zinc oxide thin film transistors. *Appl. Phys. Lett.* **2008,** *93* (20), 203506.

(5) Zhang, Y.; Li, J.; Zhang, Y.; Yang, H.; Guan, Y.; Chan, M.; Lu, L.; Zhang, S. Deep Sub-Micron Self-Aligned Bottom-Gate Amorphous InGaZnO Thin-Film Transistors With Low-Resistance Source/Drain. *IEEE Electron Device Lett.* **2023,** *44* (8), 1300-1303.

(6) Shimura, Y.; Nomura, K.; Yanagi, H.; Kamiya, T.; Hirano, M.; Hosono, H. Specific contact resistances between amorphous oxide semiconductor In–Ga–Zn–O and metallic electrodes. *Thin Solid Films* **2008,** *516* (17), 5899-5902.

(7) Bae, S. H.; Ryoo, H. J.; Yang, J. H.; Kim, Y. H.; Hwang, C. S.; Yoon, S. M. Influence of Reduction in Effective Channel Length on Device Operations of In-Ga-Zn-O Thin-Film Transistors With Variations in Channel Compositions. *IEEE Trans. Electron Devices* **2021,** *68* (12), 6159-6165.

(8) Bae, S.-H.; Yang, J.-H.; Kim, Y.-H.; Kwon, Y. H.; Seong, N.-J.; Choi, K.-J.; Hwang, C.-S.; Yoon, S.-M. Roles of Oxygen Interstitial Defects in Atomic-Layer Deposited InGaZnO Thin Films with Controlling the Cationic Compositions and Gate-Stack Processes for the Devices with Subμm Channel Lengths. *ACS Appl. Mater. Interfaces* **2022,** *14* (27), 31010-31023.
